# Multi-parameter control of photodetection in van der Waals magnet CrSBr

**DOI:** 10.1038/s41377-024-01737-2

**Published:** 2025-02-03

**Authors:** Shiqi Yang, Zhigang Song, Yuchen Gao, Leyan Huang, Xinyue Huang, Pingfan Gu, Wenjing Liu, Zuxin Chen, Yu Ye

**Affiliations:** 1 State Key Laboratory for Mesoscopic Physics and Frontiers Science Center for Nano-optoelectronics, School of Physics, Peking University, Beijing, 100871 China; 2 John A. Paulson School of Engineering and Applied Sciences, Harvard University, Cambridge, MA 02138 USA; 3 Academy for Advanced Interdisciplinary Studies, Peking University, Beijing, 100871 China; 4 School of Semiconductor Science and Technology, South China Normal University, Foshan, 528225 China; 5 Collaborative Innovation Centre of Quantum Matter, Beijing, 100871 China; 6 Yangtze Delta Institute of Optoelectronics, Peking University, Nantong, 226010 Jiangsu China; 7 Liaoning Academy of Materials, Shenyang, 110167 China

**Keywords:** Electronics, photonics and device physics, Physics

## Abstract

Photodetectors equipped with multi-parameter control hold the potential to deliver exceptional performance in a wide range of scenarios, paving the way for developing novel spin-opto-electronic devices. Nevertheless, the integration of such capabilities within a single device is challenging due to the necessity of harmonizing multiple materials with varying degrees of freedom. In this study, we introduce the van der Waals magnet CrSBr, featuring inherent anisotropy and distinctive spin-electronic coupling, to this realm. The linear dichroic ratio of the photocurrent in CrSBr tunneling device can reach ~60 at 1.65 K, and the photoresponse experiences a significant boost with increasing magnetic field. Additionally, the unique spin-charge coupling engenders a photon energy-dependent photocurrent that is modulated by an external field and is validated by first-principle calculations. Our findings elucidate the effective multi-parameter control of photodetection based on vdWs magnet CrSBr, highlighting its potential applications in cutting-edge optoelectronic devices and as a highly sensitive probe medium.

## Abstract (web summary)

The CrSBr photodetector exhibits photon energy-dependent photocurrent modulated by external magnetic field and light polarization, offering promise for advanced spin-optoelectronic devices.

## Graphical abstract

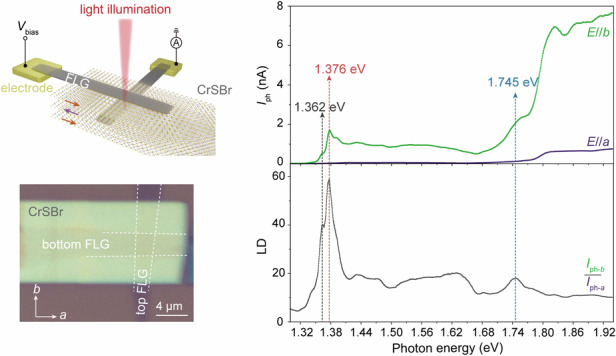

## Introduction

Multi-parameter control of the optoelectronic properties, involving variables such as photon energy, polarization, and external magnetic field, greatly improves the functionality and adaptability of devices such as photodetectors^1–9^. Different parameters add multiple degrees of freedom to photodetection and can be used to carry different data streams for encoding, potentially multiplying the capacity. At the same time, photodetectors can be made highly sensitive to specific parameters by applying various external manipulation approaches, which is critical to improved sensitivity, data capacity, and communication speed to facilitate its applications from sophisticated imaging systems to quantum computing and cryptography^10–16^. However, each parameter often requires a separate control mechanism, leading to complicated designs that integrate the ability to control and modulate these parameters into a single device^6–9^. The fundamental obstacle to achieving high sensitivity across these parameters is finding and developing materials that respond simultaneously well to external fields and light illuminations. Given the current technological and material limitations, the complexity of achieving such control poses a significant challenge to cutting-edge research in this field.

**Fig. 1:**
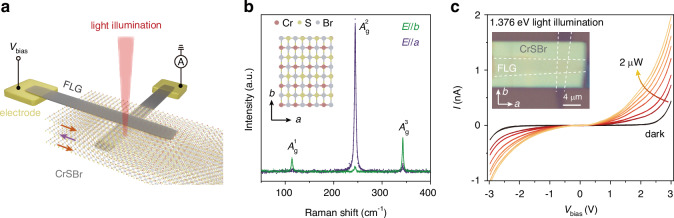
**CrSBr photodetector based on vertical junction**. **a** Schematic illustration of a CrSBr photodetector in its ground AFM state, with top and bottom FLG contacts, and light illumination. **b** Raman spectra of a CrSBr flake at room temperature under co-polarized excitation and collection measurement conditions with the laser polarized along the *b* and *a* axes. ${A}_{g}^{1}$ and ${A}_{g}^{3}$ modes exhibit maximum intensity when polarized along the *b* axis, while the ${A}_{g}^{2}$ mode shows maximum intensity when polarized the *a* axis. Inset shows the in-plane atomic crystal structure of CrSBr. **c** The *I*-*V* curves of a 47-nm CrSBr device (D1) under dark condition and with increasing power of 1.376-eV light illumination at 1.65 K, indicating a significant photocurrent response. Inset shows the optical image of D1

Recently discovered two-dimensional (2D) magnetic semiconductor CrSBr provides an extraordinary platform for coupling the electronic and optical properties with external multiple parameters. It is a direct bandgap layered semiconductor formed by an orthorhombic structure with *P*_*mmn*_ (*D*_2*h*_) space group^17–20^. The crystallographic *b* and *a* axes of the elongated exfoliated CrSBr flake correspond to its short and long edges, respectively, revealing the innate anisotropy of this material (inset in Fig. 1b). Another intriguing feature of CrSBr is its pronounced magneto-optical response. By applying an external magnetic field along the hard magnetization *c*-axis, the spin configurations will evolve from an in-plane A-type antiferromagnetic (AFM) state to an out-of-plane ferromagnetic (FM) state, resulting in a drastic change of the optical transitions due to the spin-allowed interlayer hybridization^20–25^. Moreover, the in-plane strain and hydrostatic pressure applied also show a prominent tuning of its magnetic properties^26,27^, indicating a sensitive and broad response to external fields. Leveraging its strong anisotropy and magneto-electronic coupling effects, CrSBr is emerging as a promising candidate for exploring high-performance spin-opto-electronic devices that can be effectively controlled by an external magnetic field, illuminated photon energy, and polarizations.

## Results

To explore the photoresponse of CrSBr, a vertical van der Waals (vdWs) heterostructure was assembled, comprising two few-layer graphene (FLG) strips clamping a CrSBr nanoflake, as depicted in Fig. 1a. Vertical junction devices with CrSBr thicknesses varying 21 nm, 47 nm, and 80 nm were fabricated and examined, producing closely comparable results (Fig. S1). Throughout the following discussion, the focus centers on the device featuring a CrSBr thickness of 47 nm (referred as D1) and the corresponding experimental findings. Initially, polarized Raman spectroscopy was utilized to ascertain the crystallographic orientation of the CrSBr flakes as shown in Fig. 1b. In the co-polarized measurement configuration, the ${A}_{g}^{1}$ and ${A}_{g}^{3}$ modes exhibited maximum intensity when the laser excitation was polarized along the *b* axis, while the ${A}_{g}^{2}$ mode displayed maximum intensity when the excitation was polarized along the *a* axis^20^. In this study, our measurements were primarily conducted at 1.65 K. Under dark conditions, the current-voltage (*I–V*) characteristics of the heterostructure showed pronounced tunneling behavior (Fig. 1c), indicating insulating behavior at low voltages and conducting current above 2.5 V, suggesting adherence to the Fowler-Nordheim tunneling model^28–30^. Upon illumination with light (with a spot diameter of ~1 µm) at an energy of 1.376 eV polarized along the *b* axis (*E*//*b*) at the junction region, a significant increase in current was observed (Fig. 1c). Notably, the vertical vdWs heterostructure exhibited enhanced photodetection sensitivity (see comparisons in Fig. S1) and response speed compared to the planar configurations due to the shorter transit lengths of photogenerated charge carriers^31–33^. The photocurrent demonstrated a monotonic increase with the bias voltage (Fig. S2), with the device’s photodetection characteristics primarily analyzed at a bias voltage of 2.7 V. The measured photocurrent under light illuminations (*I*_ph_ = *I*_light_−*I*_dark_) exhibited a linear relationship with incident laser power within 2 µW, resulting in an extracted photoresponsivity (*R*, defined as the ratio of photocurrent to illumination power) of ~0.8 mA W^-1^ (Fig. S2).

**Fig. 2:**
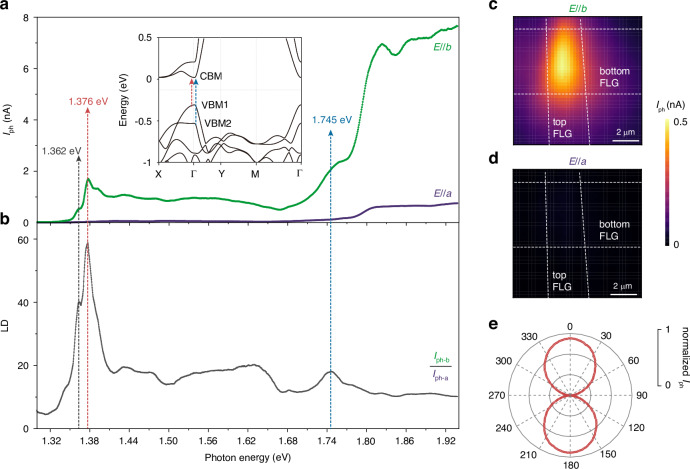
**Photon energy- and polarization-dependent photocurrent**. **a** Photon energy-dependent photocurrent under 2-*µ*W incident light polarized along the *b* axis (green) and *a* axis (violet) at 1.65 K, showing significant anisotropic photocurrent over a wide spectral range from 1.30 eV to 1.93 eV. Inset depicts the band structure of bilayer CrSBr from a first-principle calculation, showing a flat conduction band along the Γ-X direction corresponding to the *a* axis, and dispersive bands along the Γ-Y direction corresponding to the *b* axis. Red and blue dashed arrows indicate the interband transitions from VBM1 to CBM and VBM2 to CBM. **b** Linear dichroic ratio defined by *I*_ph*−*b_*/I*_ph*−*a_, showing a high degree of polarization-sensitive response and prominent peaks at interband transitions (indicated by the dashed arrows). **c**, **d** Spatial mappings of photocurrent signal measured from D1 with light polarization along the *b* axis (**c**) and *a* axis (**d**). **e** Polarization-angle dependence of the photocurrent with incident light of 1.376 eV plotted in polar coordinates, where angle 0^°^ (90^°^) corresponding to the *b* axis (*a* axis) of CrSBr

Derived from Cr-S chains along the *b* axis and weak interlayer hybridization, the vdWs semiconductor CrSBr exhibits strong one-dimensional electronic properties, as well as anisotropic exciton behavior^34^. This promotes CrSBr as a promising medium to manifest unique photon energy-polarization-dependent properties for photodetectors. Here, we utilize a super-continuous laser combing with a half-wave plate to probe the photocurrent response with photon energy ranging from 1.30 eV to 1.93 eV. When the incident light is polarized along the *b* axis, the *I*_ph_ exhibits a sharp peak at 1.376 eV (Fig. 2a, green line). The direct interband transition from the valence band maximum (VBM1) to the conduction band minimum (CBM) of CrSBr (inset in Fig. 2a) at the Γ point only allows the absorption of photons polarized along the *b* axis^19–23,34–36^. Therefore, we infer that the peak at 1.376 eV in *I*_ph_ corresponds to the excitonic effect of interband transition from VBM1 to the CBM, which is also consistent with the peak inferred from the differential reflection spectra^20–23^. The experimental verification of the absorption peak at 1.362 eV remains uncertain, and it is speculated to arise from predicted excitonic resonances^34^, warranting further exploration. Weak peaks below 1.362 eV may be related to the prevalent Cr and Br vacancies in the sample^36^. We observe several peaks in the high-energy region around 1.376 eV, and similarly, several peaks above the excitonic peak are also observed in the photoluminescence (PL) spectrum (Fig. S3). These peaks above the interband transition may be due to the brightening of the otherwise forbidden optical transition from the VBM1 to the second local minimum conduction band, induced by asymmetric dielectric environments (such as the asymmetry between the top and bottom FLG) or phonon-assisted indirect transitions^34^. The photocurrent remains almost constant for incident photon energies between 1.50 eV and 1.68 eV. Another peak in *I*_ph_ appears when the incident photon energy reaches 1.745 eV, which corresponds well to the optical transition from the second valence band maximum (VBM2) to the CBM, with the VBM2 located approximately 0.4 eV below the VBM1 (inset of Fig. 2a). At a 1.920-eV light illumination, the photoresponsivity of the CrSBr photodetector could reach 4 mA W^−1^ (Fig. S4), which is higher than typical photodetectors based on intrinsic 2D materials^37–39^. By contrast, when the incident light is polarized along the *a* axis, only a weak photocurrent signal is detected in the whole energy region (Fig. 2a, violet line). From the photocurrent spatial maps, a significant photocurrent signal is observed in the overlapping region of the top and bottom FLG under illumination at 1.376 eV with *E//b*, while no detectable photocurrent is observed throughout the device region with *E//a* (Fig. 2c, d).

Based on the highly anisotropic absorption and the resulting linearly polarized light-dependent photoresponse in CrSBr device, we can define the linear dichroic (LD) ratio spectrum of the CrSBr photodetector by comparing the photocurrent along *b* and *a* axes (*I*_ph*−*b_*/I*_ph*−*a_, as shown in Fig. 2b). At an incident photon energy of 1.376 eV, the LD ratio can reach about 60, which is superior to the polarization-sensitive photodetectors based on 2D intrinsic materials so far, most of which maintain LD values below 10^14,40–46^. The stronger LD ratio can be attributed to the enhanced anisotropic electronic band structure combing with its optical selection rules^21,34^, as well as the more efficient photocurrent collection in the CrSBr vertical photodetector structure. The polar plot of the photocurrent dependence on the incident light polarization angle further confirms the highly anisotropic photocurrent response (Fig. 2e, see more in Fig. S5). In fact, in addition to its promising use as a polarization-sensitive photodetector, the energy-dependent LD spectra can also be utilized to delicately describe the interband transitions. In particular, due to the anisotropy of each interband transition, the LD spectra will show a drastic change as the incident photon energy crosses each optical transition, as indicated by the dashed arrows in Fig. 2a, b. It is worth noting that the LD nature of the photocurrent arises from the anisotropy of the electronic band structure due to the crystal symmetry, and should theoretically maintain at higher temperatures. Changes in the magnetic order also do not change the robust anisotropic behavior of the photocurrent (see more details in Fig. S5).

**Fig. 3:**
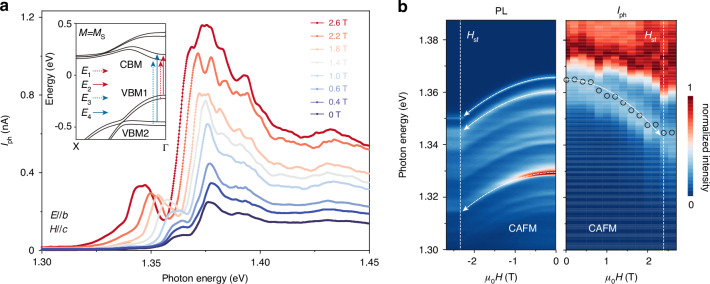
**Magnetic field-controlled photocurrent**. **a** Photon energy-dependent photocurrent under different external magnetic fields (*H*//*c*) illuminated by 0.5-µW light polarized along the *b* axis at 1.65 K. The inset is the calculated band structure of bilayer CrSBr in the FM state, and the allowed interband transitions are identified. **b** Normalized PL (left) and photocurrent (right) signals in the magnetic field-photon energy parameter space. The white dashed arrow lines are the corresponding quadratic fit to experimental data below the saturation field for *I*_ph_ peak ~1.362 eV and PL peaks. *H*_sf_ represents the spin-flip transition field that separates the canting AFM (CAFM) state and the forced FM state

The unique spin-electronic coupling in CrSBr, manifested in particular by the evolution of the electronic structure induced by the interlayer magnetic order, will lead to the continuous control of the photocurrent in the CrSBr device under an external magnetic field. This effect is extremely challenging due to the very weak magneto-optic coupling in conventional semiconductors^3^. Fig. 3a illustrates the evolution of the photon energy-dependent *I*_ph_ as a function of external magnetic field parallel to the *c* axis (see more data in Fig. S6). At first glance, the *I*_ph_ increases significantly with increasing magnetic field, which is mainly due to the reduced magnetic resistance that the photogenerated carriers encounter when the magnetic state is aligned with the external field (Fig. S7). Furthermore, the *I*_ph_ curves with photon energy experiences a continuous redshift with the increase of the applied magnetic field until it saturates at ~2.4 T. The evolution of the *I*_ph_ peak position with the magnetic field closely mirrors the changes in the PL spectrum (see Fig. 3b). The energy shift occurs within the spin-flip field (*H*_sf_) of 2.4 T confirmed by the reflective magnetic circular dichroism (RMCD) microscopy (Fig. S7). According to the perturbation theory that takes into account interlayer hopping terms^21^, the energy shift is quadratically related to *µ*_0_*H* as depicted by the white dashed arrow lines in Fig. 3b (see extracted peak values and quadratic fitting in Fig. S6).

We then performed first-principle calculations to investigate the evolution of the electronic structure of the bilayer CrSBr from the AFM state to the FM state to obtain detailed information regarding the spin angle dependence of the bandgap. In the absence of a magnetic field, the conduction and valence bands of AFM CrSBr are spin-degenerate, while in the FM state, a significant splitting in the conduction and valence bands is observed (Fig. 3a, inset). The evolution of the optical transitions from VBM to CMB can be elucidated by a function of the magnetization *M* along the *c*-axis, expressed as *M* = *M*_*s*_cos(*θ/*2), where *M*_*s*_ denotes saturation magnetization of each layer, and *θ* represents the angle between the magnetization vectors of the two layers (see detailed calculation results in Fig. S8). The calculated redshift of the *E*_1_ transition from AFM state to the FM state is 12.0 meV. The splitting of the degenerate bands with increasing magnetic field results in a wider band dispersion and absorption peak at 1.376 eV. This value is also in consistent with the 18.0 meV redshift of the *I*_ph_ peak at 1.362 eV observed in Fig. 3b, which may indicate the peak at 1.362 eV possess the similar optical transition as that at 1.376 eV. For the high-energy photocurrent response, we also observe a redshift behavior of the *I*_ph_ peak near 1.745 eV, with a redshift of up to 80 meV from the AFM to FM state (Fig. S6). The redshift of the peak position at high energies exceeds the theoretical prediction of the energetic shift (10.4 meV) of the VBM2 to CBM transition with increasing *M*, which may be due to the fact that more Cr *d*_*z*_ orbitals with out-of-plane properties are mixed in the observed high-energy optical transitions^34^. The correlation between *I*_ph_, the magnetic field, and the photon energy underscores the role of the photocurrent as a discerning tool for monitoring both the magnetic field and the electronic structure transitions induced by the magnetic field (see more discussions in Fig. S9).

**Fig. 4:**
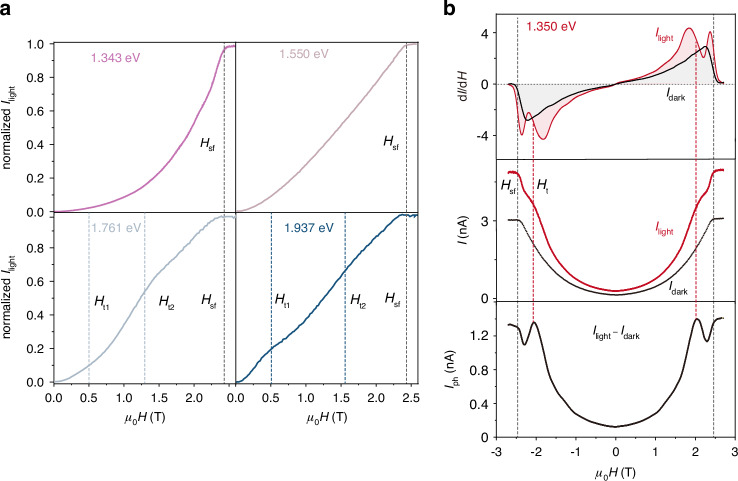
**Magneto-photocurrent at specific incident photon energies**. **a** *I*_light_ *versus µ*_0_*H* under 1.343-eV, 1.550-eV, 1.761-eV, and 1.937-eV laser excitations polarized along the *b* axis. Currents are normalized for clarity. *H*_t_ represents the magnetic field when the redshift of the optical transition energy caused by the spin-canting process spans the energy level of the incident photons. **b** Photocurrent *versus µ*_0_*H* under laser excitation of 1.350 eV. The top two panels show the *I*_light_ and *I*_dark_ signals with varying magnetic fields and their corresponding first derivatives, revealing a two-step transition positioned at *H*_t_ of 2 T and *H*_sf_ of 2.4 T. The bottom panel shows the *I*_ph_ signal, displaying a photocurrent kink at 2 T. The transition fields are determined by the steepest drops in the current derivative

Due to the intertwined coupling of spin configuration and incident photon energy in the photodetector, we are able to design magnetically dependent photocurrents with different profiles depending on the photon energy (Figs. 4a and S10). When the incident photon energy is 1.343 eV, only involving the lowest-energy absorption under all external magnetic fields, the *I*_light_ exhibits a parabolic increase and saturation at *H*_sf_ with the magnetic field, consistent with the dark current profile. At an incident photon energy of 1.550 eV, involving the optical transition from VBM1 to CBM without any extra absorption under the magnetic field, the *I*_light_ smoothly varies with the magnetic field and shows near-linear behavior over a wide range of magnetic fields (Fig. 4a). The above smooth magnetic field dependence allows us to detect small variations in the magnetic field using *I*_light_. Under a magnetic field variation of 10 mT, *I*_light_ accurately reflects the magnetic field variation curve with stable and reproducible responses (Fig. S10). By contrast, when the incident photon energy is set at 1.350 eV, the optical transition of the VBM1 to CBM redshifts with the magnetic field, and will cross the energy level of 1.350 eV at around 2 T, leading to a two-step evolution of *I*_light_ with the magnetic field (Fig. 4b, *H*_t_ and *H*_sf_). The first derivative spectrum of the *I*_light_ (*I*_dark_) current with respect to the magnetic field shown in the top panel of Fig. 4b reveals two (one) prominent peaks, indicating the two-step (one-step) transitions. Interestingly, *I*_ph_ also shows kinks near ±2 T before the saturation at *H*_sf_ (Fig. 4b, bottom panel), which might be due to the joint effect of the involvement of additional optical transitions and the increasing tunneling (dark) current under external magnetic field of the device. For high-energy incident photons, *I*_light_ shows significant multi-step evolutions, e.g., for 1.761-eV incident photon, the kinks are around ±0.5 and ±1.3 T, and for 1.937-eV incident photon, the kinks are ~±0.5 and ±1.6 T deduced from their first differentials (Figs. 4a and S10). These transitions at *H*_t_ serve as an indication of the magnetic field required for the optical transitions in CrSBr to span the energy level of the incident photon.

It also should be noted that the breaking of the time-reversal symmetry will result in nonzero circular dichroism, which can also be used for the detection of magnetic orders under an external magnetic field^15,16,30^. As shown in Fig. S11, the difference of the photocurrent in CrSBr device under illumination by the right- and left-handed circularly polarized light exhibits a continuous increase from 0 to 3 nA with increasing field from 0 T to 2.4 T, followed by a saturation behavior. This is in excellent agreement with the RMCD and magnetoresistance measurements displaying a spin canting process saturated at 2.4 T (Fig. S7). The photocurrent helicity, defined as *P* = [*I*_ph_(*σ*_+_)−*I*_ph_(*σ*_*−*_)]*/*[*I*_ph_(*σ*_+_)+*I*_ph_(*σ*_*−*_)], reaches ±7.4% at the saturation field.

## Discussion

In summary, our investigation delved into the dependence of photocurrent on photon energy, polarization (linear and circular), and magnetic field in CrSBr vertical junction devices, demonstrating the adept control of photodetection through multiple parameters. The measurements revealed a remarkable LD ratio and a substantial response to magnetic fields, due to the robust anisotropic electronic band structure and the magneto-electronic coupling effect. The observed redshift of the photocurrent peaks and the enhanced photoresponsivity with magnetic field underscore the intricate interplay between photocurrent, electronic structure, optical transitions, and magnetic order. The magneto-photocurrent exhibited by CrSBr shows promise for potential applications in magnetic sensing, and presents a unique potential as an “on/off” switch in response to changes in external magnetic fields. The integration of CrSBr with other magnetic materials opens avenues for probing magnetic domain structures through spatial mapping of the photocurrent. Furthermore, the identification of anomaly points in the photocurrent with magnetic field variations establishes its efficacy as a robust tool for probing the evolution of electronic structures at specific energy levels. Lastly, the concurrent response of the photocurrent to both linear and circularly polarized light under external fields positions CrSBr as an intriguing platform for exploring the potential spin-opto-electronics devices.

## Materials and methods

### Crystal synthesis and device fabrication

CrSBr single crystals were grown using the chemical vapor transport method. Disulfur dibromide and chromium metal were mixed in a molar ratio of 7:13 and then sealed in a silica tube under vacuum. The evacuated silica tube was then placed in a two-zone tube furnace. CrSBr crystals were grown under a temperature gradient from 950 °C to 880 °C for 7 days, with a heating/cooling rate of 1 °C/min. FLG and thin flakes of CrSBr were mechanically exfoliated on polydimethylsiloxane. The devices were assembled layer by layer using a dry transfer technique onto prepatterned electrodes on a 285 nm Si/SiO_2_ substrate, with the top/bottom graphene flakes connected to the prepatterned electrodes. Transparent FLG were utilized here so that light can be absorbed by the semiconductor CrSBr layer to generate photocurrent. The sample thickness was determined by atomic force microscopy under ambient conditions.

### Magneto-optoelectronic measurements

The magneto-optoelectronic measurements were conducted using the Attocube closed-cycle cryostat (attoDRY2100) with a base temperature of 1.6 K and a maximum out-of-plane magnetic field of 9 T. For current measurements, an electrometer (Keithley 2636B) served as a DC voltage source and ammeter. Photocurrent spatial mapping was obtained by scanning the piezo voltage to move the sample. Anisotropic photocurrent measurements were performed by sweeping the polarization angle of the illumination light. The direction of the electric vector of the light was adjusted with respect to the crystal *b* axis by inserting a *λ*/2 plate in the optical path. The angle was set to be 0^°^ when the light polarization was along the *b* axis for clarity. For the helicity dependence of the photocurrent, an achromatic quarter-wave plate was introduced into the optical path to produce different circularly polarized light. Photon energy-dependent measurements were carried out using a super-continuum laser (SuperK FIANIUM FIU-15) spanning 1.3 eV to 1.94 eV. The data presented in Fig. 3a have been smoothed, and the original data are provided in the Supplementary Information. PL measurements were conducted in a reflection geometry, using the same objective for the excitation and collection of the reflected light. The light collected from the sample was directed to a monochromator with a grating of 600 grooves/mm (HRS500S) and detected using a liquid nitrogen-cooled camera (PYL-400BRX). The RMCD measurement utilized a He-Ne 633-nm laser with quarter-wave modulation from a photoelastic modulator at 50.02 KHz and a chopper at 790 Hz. The reflected signal was captured by a photomultiplier tube and processed by a two-channel lock-in amplifier (Zurich HF2LI).

### First-principle calculations

First-principle calculations were performed using VASP. The basis set of projector-augmented plane waves with a cutoff of 400 eV was employed. The PBE functional was utilized to account for the exchange-correlation interaction. Spin-orbit coupling was enabled to incorporate nonlinear spin effects. A vacuum space exceeding 15 °A was introduced in directions vertical to the material plane to decouple the periodic images. All atoms and lattice parameters were relaxed until the force on each atom was less than 0.01 eV/°A.

## Supplementary information

Supplementary Information

## Data Availability

All relevant data are available in the main text, Supplementary Information, or upon request to the authors.
