# Supplementary material for: Multi-parameter control of photodetection in van der Waals magnet CrSBr
Source: Light Sci Appl. 2025 Feb 3;14:67. doi: 10.1038/s41377-024-01737-2 (PMC11790870; doi:10.1038/s41377-024-01737-2)
Supplement: Supplementary file 1 — Supplementary Information [file 41377_2024_1737_MOESM1_ESM.pdf]

Supplementary Information for
Multi-parameter control of photodetection in van der Waals magnet CrSBr

Shiqi Yang[1], Zhigang Song[2], Yuchen Gao[1], Leyan Huang[1], Xinyue Huang[1,3], Pingfan Gu[1], Wenjing Liu[1], Zuxin Chen[4] and Yu Ye[1,5,6,7,†]

[1]State Key Laboratory for Mesoscopic Physics and Frontiers Science Center for Nano-optoelectronics, School of Physics, Peking University, Beijing 100871, China
[2]John A. Paulson School of Engineering and Applied Sciences, Harvard University, Cambridge, Massachusetts 02138, United States
[3]Academy for Advanced Interdisciplinary Studies, Peking University, Beijing 100871, China
[4]School of Semiconductor Science and Technology, South China Normal University, Foshan 528225, China
[5]Collaborative Innovation Centre of Quantum Matter, Beijing 100871, China
[6]Yangtze Delta Institute of Optoelectronics, Peking University, Nantong 226010 Jiangsu, China
[7]Liaoning Academy of Materials, Shenyang 110167, China

[†]Corresponding to: ye_yu@pku.edu.cn

This PDF file includes:
Additional experimental results, details, and methods.

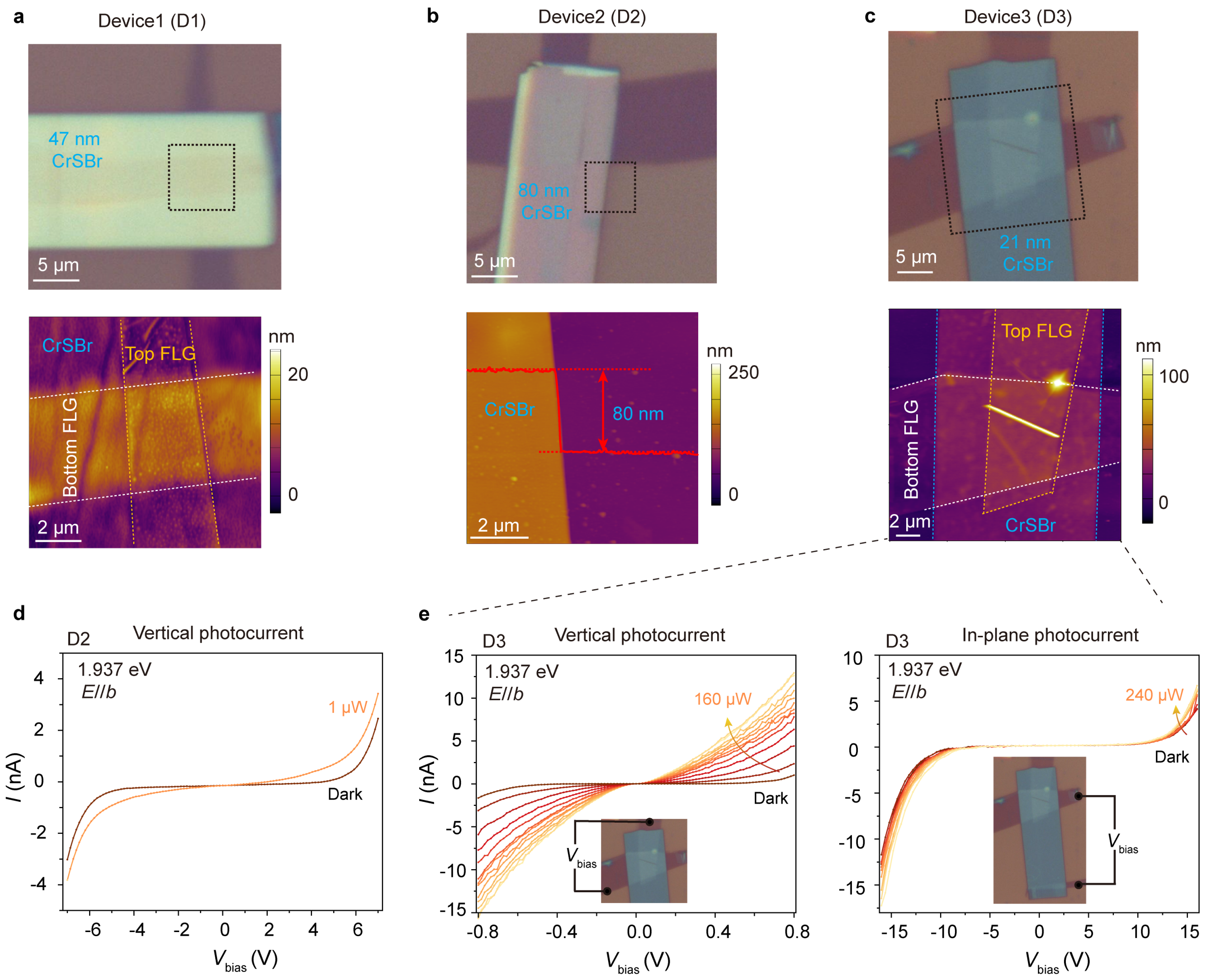

FIG. S1. **Characterization of devices with different CrSBr thicknesses.** (a-c) Optical images and corresponding atomic force microscopy of three CrSBr juction devices with thickness of 47 nm (a), 80 nm (b), and 21 nm (c). The data in the main text are mainly from the D1. (d) $I-V$ curves of the power dependence under 1.937-eV laser excitation in D2. (e) $I-V$ curves of the power dependence under 1.937-eV of vertical and in-plane photocurrent from D3, indicating a weaker photoresponse of the in-plane structure.

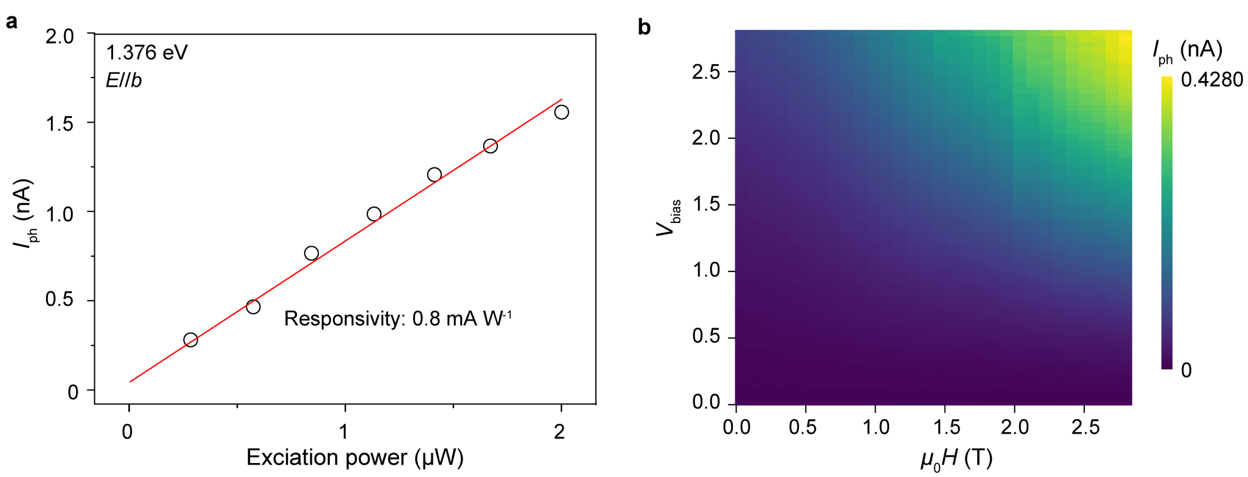

FIG. S2. **Photocurrent response of D1.** (a) The photocurrent in D1 under 1.376-eV laser excitation with power increasing from 0.28 μW to 2 μW, showing a photoresponsivity of $\sim$0.8 mA W^{-1}. (b) Photocurrent in the parameter space of bias voltage and magnetic field, showing that the photocurrent monotonically increases with increasing bias voltage and magnetic field.

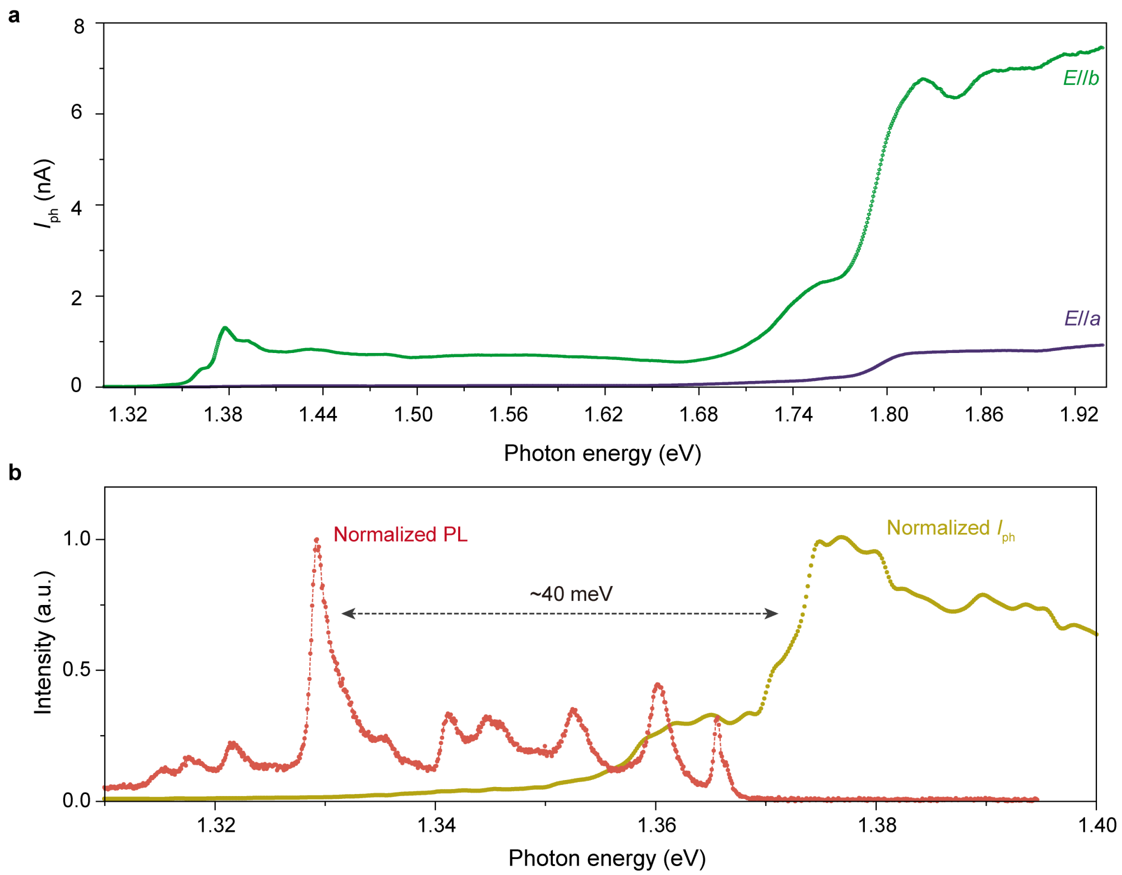

FIG. S3. **Photon energy-dependent photocurrent and PL measurements.** The normalized photocurrent (yellow) and PL spectra (red) in the CrSBr device, showing significant peaks at 1.376 eV and 1.331 eV, respectively.

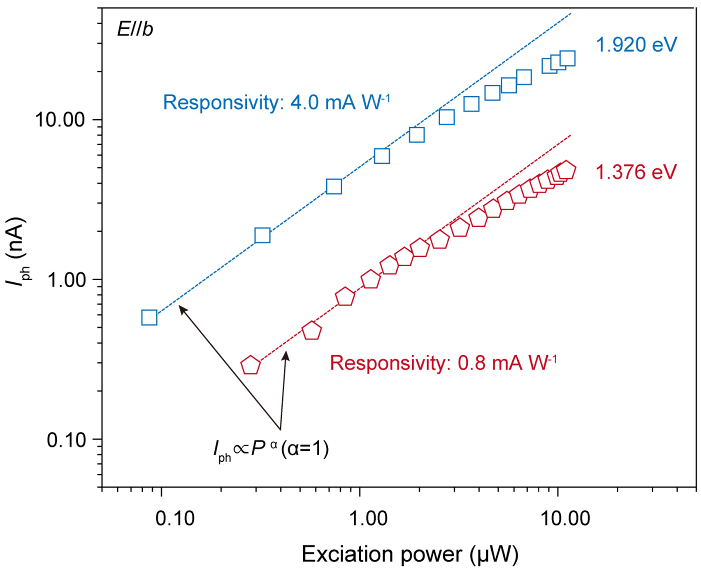

FIG. S4. **Photocurrent *versus* incident power for different incident photon energies of D1.** (a) At low incident power, the photocurrent grows linearly with incident power ($I_{\mathrm{ph}} \propto P^{\alpha}$, $\alpha = 1$), resulting in extracted photocurrent responsivities of 0.8 mA W^{-1} and 4.0 mA W^{-1} under 1.376-eV and 1.937-eV laser excitations, respectively.

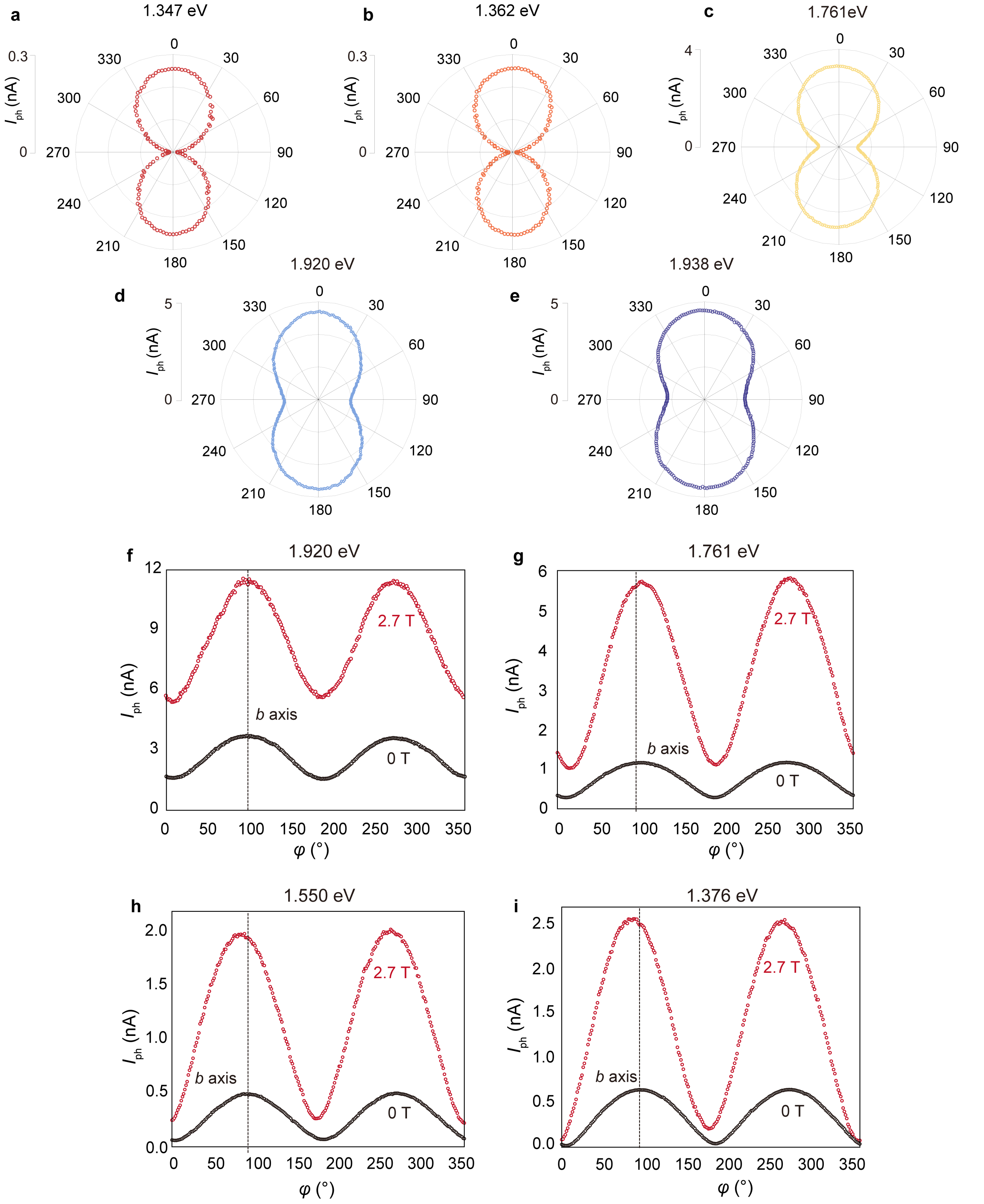

FIG. S5. **Photocurrents under different laser excitations, polarization angles, and magnetic field of D1.** (a-e) Polarization-angle dependence of the photocurrent plotted in polar coordinates at 0 T with incident light of 1.376 eV, 1.362 eV, 1.761 eV, 1.920 eV, and 1.938 eV with power of 1 μW. (f-i) Anisotropic behavior incident light of 1.920 eV, 1.761 eV, 1.550 eV, and 1.376 eV, showing almost constant anisotropic polarization. We note that under certain photon energy excitations, the measured I_{ph} maxima at 2.7 T show an angular rotation of $\sim 8°$ compared to that at 0 T, which may be due to the Faraday effect of the objective lens in the presence of a magnetic field.

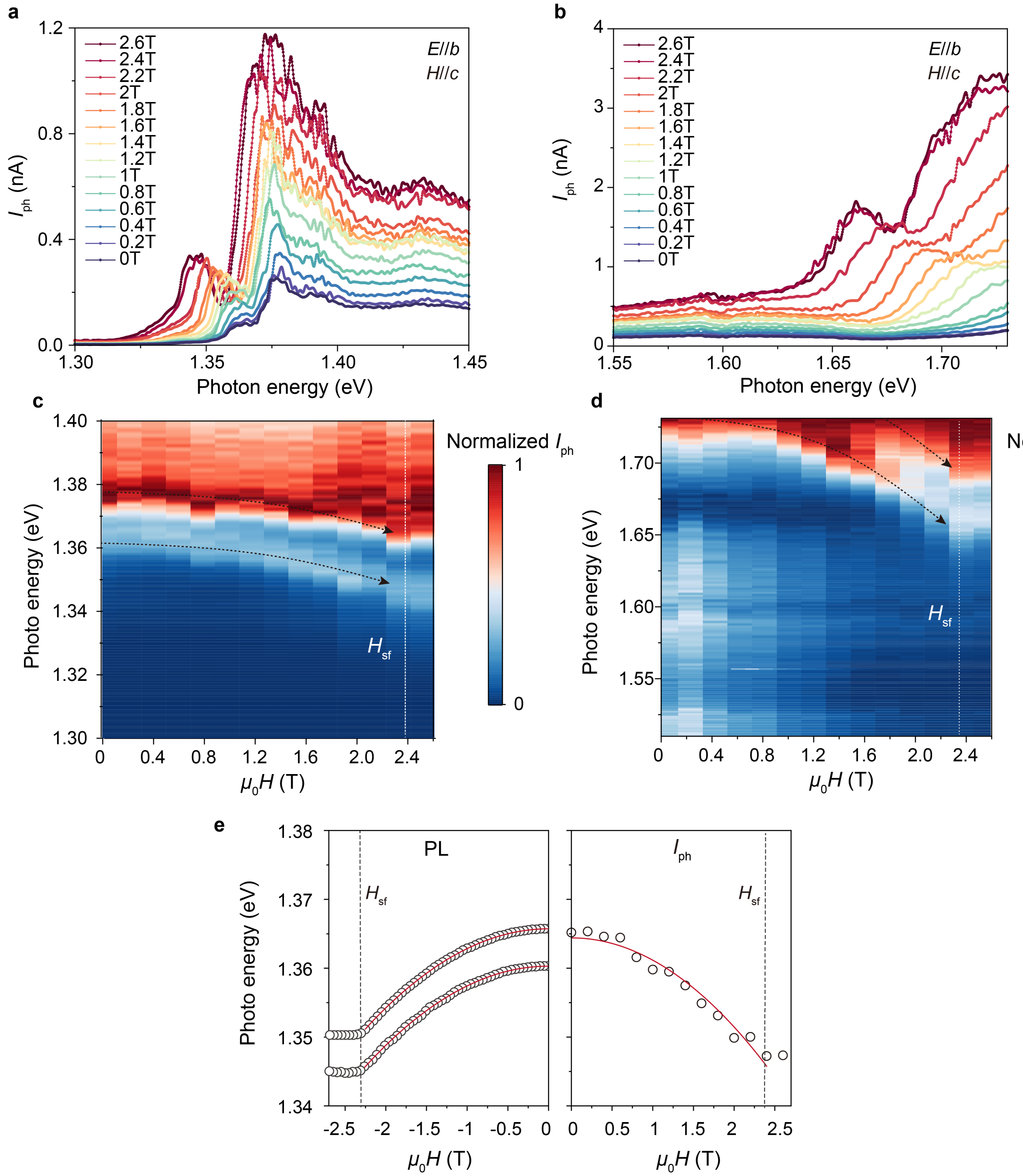

FIG. S6. **Magnetic field-controlled photocurrent.** (a-b) Photon energy-dependent photocurrent under different external magnetic fields parallel to the c axis illuminated by 0.5-μW laser excitation polarized along the b axis in the photon energy range of 1.30-1.45 eV (a) and 1.55-1.73 eV (b). (c-d) Normalized photocurrent in the magnetic field-photon energy parameter space in each range. The black dashed arrow lines indicate the redshift of each absorption peaks. The $H_{\rm sf}$ represents the spin-flip transition field that separates the CAFM state and the forced FM state. The two peaks in (c) are redshifted by $\sim$18 meV, and the peak in (d) redshifted by more than 80 meV. The energy remains almost constant when the magnetic field exceeds 2.4 T, which is consistent with spin-flip field of CrSBr along the c axis. (e) Extracted values and quadratic fit curves for each peak in PL and photocurrent measurements.

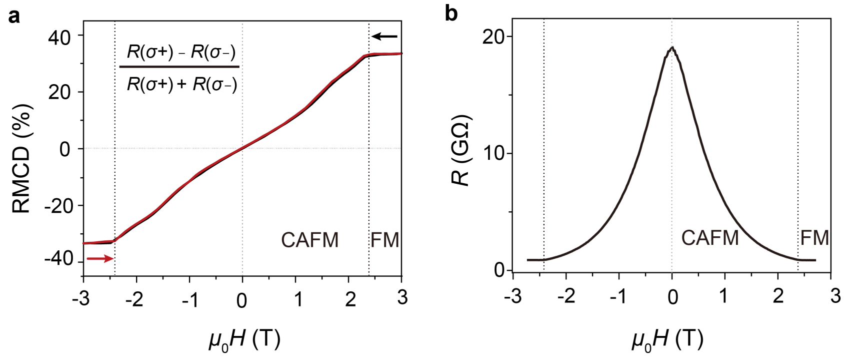

FIG. S7. **RMCD and magnetoresistance charaterization of D1.** (a) RMCD signal as a function of $\mu_0 H$ swept along the c axis. (b) Magnetoresistance of CrSBr D1 in dark environment as a function of $\mu_0 H$ swept along the c axis.

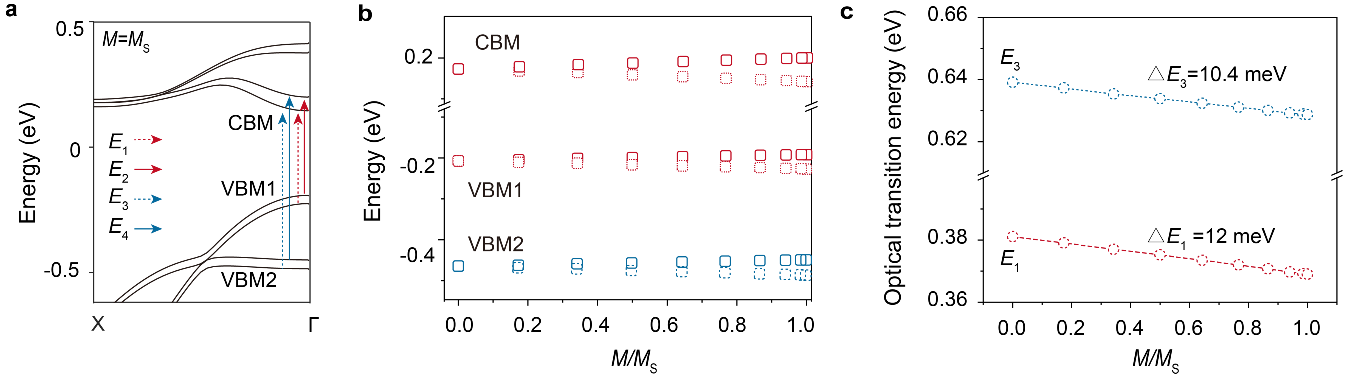

FIG. S8. **DFT calculations of the electronic structure from in-plane A-type AFM state to out-of-plane FM state.** (a) Calculated band structure of bilayer CrSBr in the FM state, and the allowed interband transitions are identified. (b) Calculated energy level evolution of CBM, VBM1, and VBM2. M is the magnetization along the c axis, which is defined as $M = M_s \cos(\theta/2)$, where M_s is the saturation magnetization of each layer, and θ is the angle between the magnetization vectors of the two layers. (c) Calculated optical bandgap evolution for the VBM1 to CBM (E_1) and VBM2 to CBM (E_3) interband transitions, showing decreasing values for each optical transition.

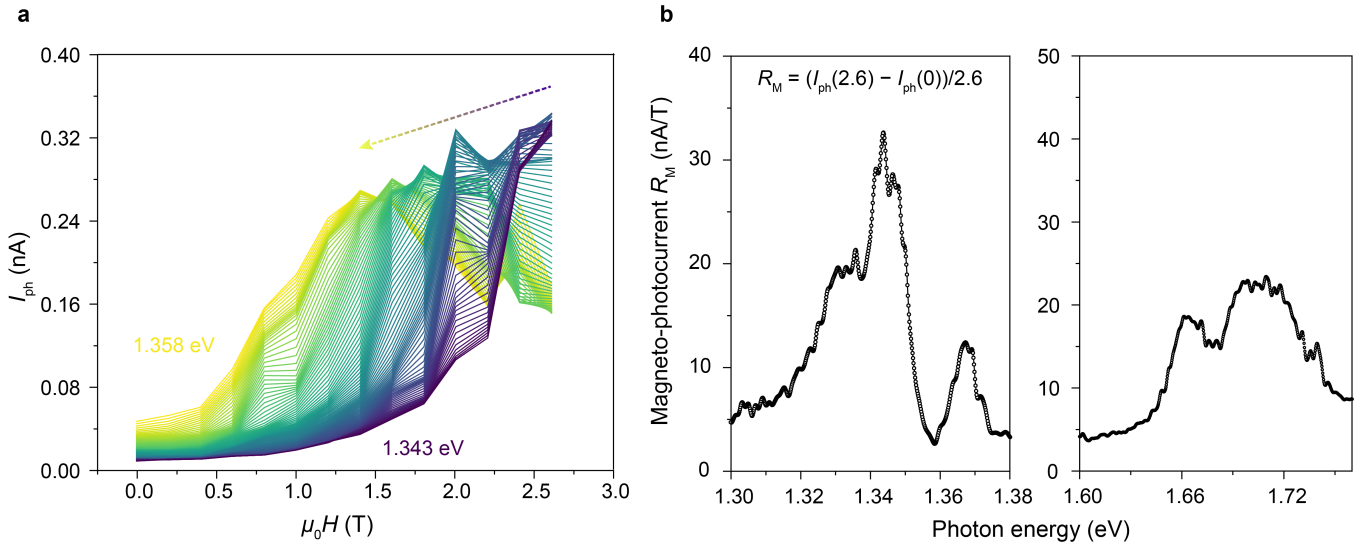

FIG. S9. **Magnetic field-controlled photocurrent of D1.** (a) Magnetic field dependence of photocurrent with incident photon energies ranging from 1.343 eV and 1.358 eV. The results are extracted from the data in Fig. S6(a). We can illustrate the different magnetic field-dependent profiles at different excitation energies for different potential applications. For example, when illuminated by a 1.343-eV laser excitation, I_{ph} increases abruptly and rapidly with magnetic field above 1.5 T, reflecting a behavior similar to the magnetic field threshold. Thus, for an incident light of 1.343 eV, setting the magnetic field to a certain value (e.g. 1.5 T) produces device low ("off" state) and high ("on" state) photocurrents within a tolerance range that corresponds to an "on/off" switch associated with the magnetic field. Illuminating the CrSBr device with photons of different energies will lead to different magnetic field response profiles. (b) The magneto-photocurrent responsivity (R_{M}) of the device ($R_{\mathrm{M}} = (I_{\mathrm{ph}(B)} - I_{\mathrm{ph}(0)})/B$) at B=2.6 T, which suggests a responsivity of 30 nA T^{-1} for 1.343 eV. Note that this value is only an indication of the response, since the photocurrent increases not linear with respect to the magnetic field.

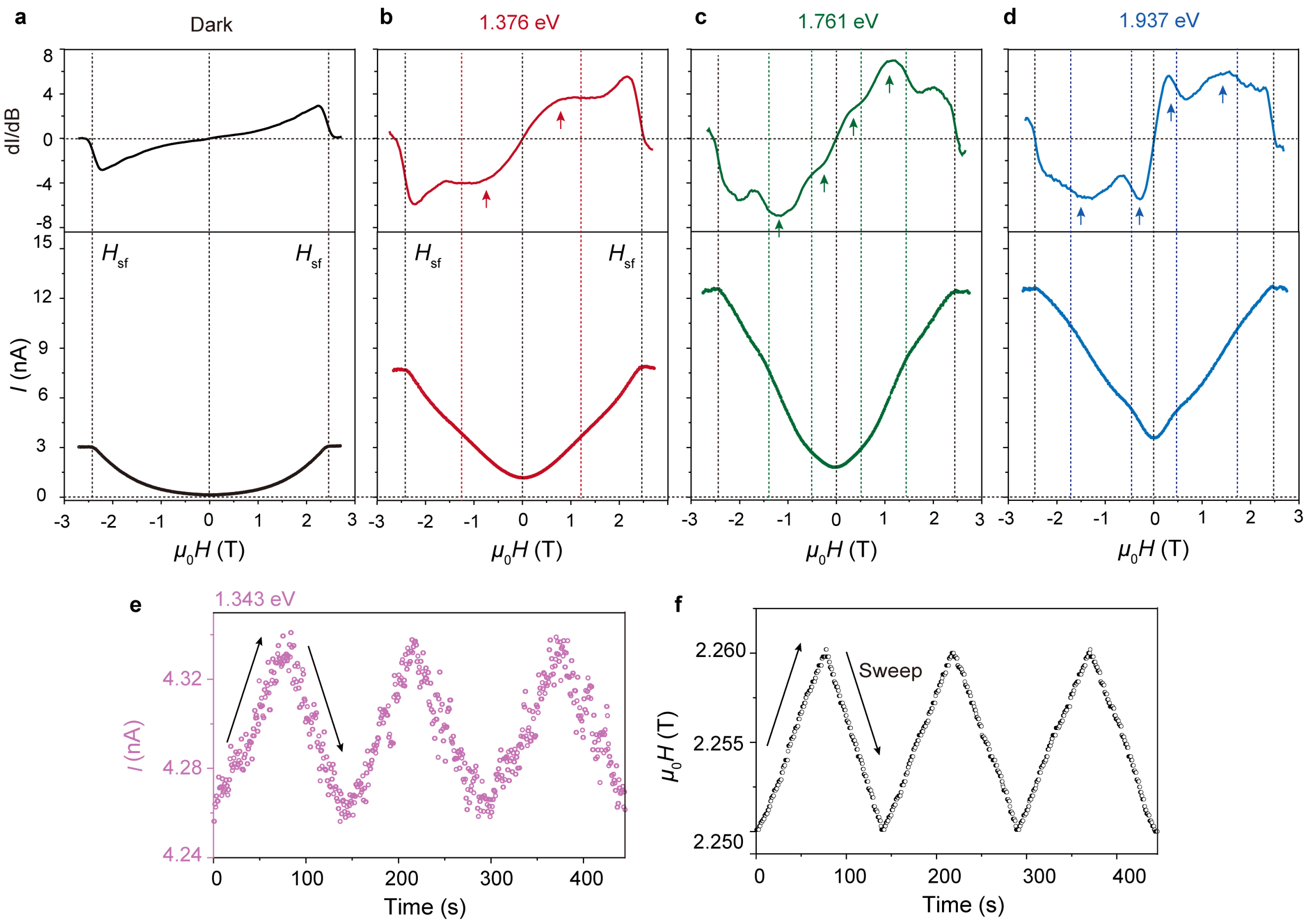

FIG. S10. **Magneto-photocurrent at specific photon energies.** (a-d) Current *versus* $\mu_0 H$ in dark (a) and light excitation of 1.376 eV (b), 1.761 eV (c), and 1.937 eV (d). The top panels show the first derivatives. Arrows indicate the additional transitions other than the spin-flip transition around 2.4 T. The transition fields H_t shown in the main text are determined by the steeply falling position of the current derivative. (e-f) The current evolution under 1.343-eV light illumination polarized along the b axis under continuous sweeping magnetic field strength ranging from 2.25 T to 2.26 T. By depicting the magnetic field-dependent photocurrent with refined field steps (mT levels), one can establish a relationship between $I_{\rm ph}$ (e) and the external magnetic field (f), which can be used to probe the changes of the magnetic field. The $I_{\rm light}$ shows a synchronized response to the magnetic field.

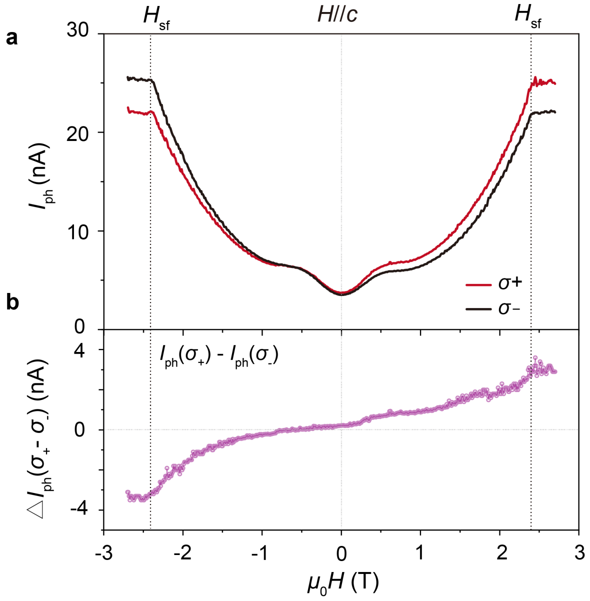

FIG. S11. **Photocurrent helicity of D2.** (a) Photocurrent response *versus* $\mu_0 H$ for different circularly polarized light illumination. (b) Variation of the circular photocurrent difference ($\triangle I_{\rm ph}(\sigma_+ - \sigma_-) = I_{\rm ph}(\sigma_+) - I_{\rm ph}(\sigma_-)$) measured from D2 with $\mu_0 H$ at a light power of 5 μW.
